# Improving Social Isolation and Loneliness Among Adolescents With Physical Disabilities Through Group-Based Virtual Reality Gaming: Feasibility Pre-Post Trial Study

**DOI:** 10.2196/47630

**Published:** 2023-12-06

**Authors:** Byron Lai, Raven Young, Mary Craig, Kelli Chaviano, Erin Swanson-Kimani, Cynthia Wozow, Drew Davis, James H Rimmer

**Affiliations:** 1 Division of Pediatric Rehabilitation Medicine Department of Pediatrics University of Alabama at Birmingham Birmingham, AL United States; 2 Dean's Office School of Health Professions University of Alabama at Birmingham Birmingham, AL United States

**Keywords:** therapy, mindfulness, play, friend, friends, friendship, lonely, loneliness, psychotherapy, peer, peers, recreation, disability, adolescent, adolescents, disability, disabled, physical disability, digital mental health intervention, youth, young adult, virtual reality, VR, gaming, depression, depressive, mental health, social, isolated, isolation, socialize, socializing, socialization, interaction, interactions, acceptability, game, games, gaming, exergame, exergames, exergaming

## Abstract

**Background:**

Adolescents with disabilities experience alarmingly higher rates of depression and isolation than peers without disabilities. There is a need to identify interventions that can improve mental health and isolation among this underserved population. Innovations in virtual reality (VR) gaming “standalone” headsets allow greater access to immersive high-quality digital experiences, due to their relatively low cost.

**Objective:**

This study had three purposes, which were to (1) examine the preliminary effects of a low-cost, home-based VR multiplayer recreation and socialization on depression, socialization, and loneliness; (2) quantify the acceptability of the program as measured by participant adherence, total play time, and exercise time; and (3) identify and describe behavioral mechanisms that affected participant engagement.

**Methods:**

This was a single-group, pre- to postdesign trial. The intervention was conducted at home. Participants were recruited from a children’s hospital. The intervention lasted 4 weeks and included 2×1-hour sessions per week of supervised peer-to-peer gaming. Participants used the Meta Quest 2 headset to meet peers and 2 coaches in a private party held digitally. Aim 1 was evaluated with the Children’s Depression Inventory 2 Short Form and the University of California, Los Angeles Loneliness Scale 20 items, which are measures of social isolation and loneliness, respectively. Aim 2 was evaluated through the following metrics: participant adherence, the types of games played, friendship building and playtime, and program satisfaction and enjoyment.

**Results:**

In total, 12 people enrolled (mean age 16.6, SD 1.8 years; male: n=9 and female: n=3), and 8 people completed the program. Mean attendance for the 8 participants was 77% (49 sessions of 64 total possible sessions; mean 6, SD 2 sessions). A trend was observed for improved Children’s Depression Inventory 2 Short Form scores (mean preintervention score 7.25, SD 4.2; mean postintervention score 5.38, SD 4.1; *P*=.06; effect size=0.45, 95% CI –0.15 to 3.9), but this was not statistically significant; no difference was observed for University of California, Los Angeles Loneliness Scale 20 items scores. Most participants (7/8, 88%) stated that they became friends with a peer in class; 50% (4/8) reported that they played with other people. Participants reported high levels of enjoyment and satisfaction with how the program was implemented. Qualitative analysis resulted in 4 qualitative themes that explained behavioral mechanisms that determined engagement in the program.

**Conclusions:**

The study findings demonstrated that a brief VR group program could be valuable for potentially improving mental health among adolescents with physical disabilities. Participants built friendships with peers and other players on the web, using low-cost consumer equipment that provided easy access and strong scale-up potential. Study findings identified factors that can be addressed to enhance the program within a larger clinical trial.

**Trial Registration:**

ClinicalTrials.gov NCT05259462; https://clinicaltrials.gov/study/NCT05259462

**International Registered Report Identifier (IRRID):**

RR2-10.2196/42651

## Introduction

For people with physical disabilities, adolescence is a critical period for school educators and health professionals to provide quality education and health services. In adolescence, people with disabilities build their self-identity and adopt vocational skills and health behaviors that increase the likelihood of living healthy and independent lifestyles as they transition into early adulthood [1-3]. Prior to COVID-19, adolescents with disabilities lagged in the development of adult life skills (eg, obtaining housing, seeking employment, developing intimate relationships, and participating in meaningful hobbies) [4] and experienced alarmingly higher rates of mental health disorders such as depression and isolation than peers without disabilities [5]. They were also far less likely to engage in social and health-enhancing physical activities [1,6-8]. Thus, there is a need for interventions that can tackle both mental health issues and low participation rates in physical activity. Physical activity is a critical behavior for improving gross motor function [9] and health conditions (eg, cardiovascular disease, pain, and fatigue) [10-12] and developing meaningful social relationships with peers.

Several reports across the globe have found that mental health and social isolation among adolescents with disabilities have worsened after the COVID-19 outbreak [13-17]. The consensus among these reports is that the loss of services (eg, therapy, school, or medical) and social interactions worsened mental health issues such as depression, anxiety, and maladaptive behaviors. Within the United States, the states within the southeast region (specifically the state of Alabama, where this study was conducted) have some of the lowest rates of health care access across the nation (as reported by the United Health Foundation) [18]. Disappointingly, we found in a recent COVID-19 study that depression, isolation, and exercise participation have substantially declined since the outbreak of COVID-19 among 101 adolescents with cerebral palsy living in Alabama [19]. This report found that basic needs were met for most families. However, 32.7% (n=33) felt down, depressed, or hopeless; 47.5% (n=48) felt little pleasure in doing things; and 64.4% (n=65) felt isolated after COVID-19. Moreover, 74.3% (n=75) reported decreased socialization and 51.5% (n=52) reported reduced exercise participation.

Recent technological innovations have made internet connectedness through virtual reality (VR) gaming far more accessible for the average consumer. In 2020, Facebook (now referred to as Meta) released the Meta Quest 2, which was the first “standalone” VR head-mounted display (HMD) designed for gaming. The headset has built-in motion tracking, a computer processor, and graphics, which allows for high-resolution gaming of up to 120 frames per second. Because of these specifications, the Quest (specifically the Quest 1) was the first HMD of its kind to not require a cable connection to a costly gaming computer. The cost of the Quest 2 (US $300) is lower than traditional gaming consoles, which has made it the first VR HMD to be incorporated into the mainstream. The Quest 2 comes with accessible handheld controllers that can be used to elicit a moderate-intensity exercise response among youth who use wheelchairs [20]. Moreover, the Quest 2 comes with preinstalled software that allows users to socialize in a digital private party via audio and digital avatars, making it a potentially valuable tool for internet wellness programs. Once a user puts on and loads into the headset, they are immediately placed within a “home” digital environment. This environment now allows the user to customize their home environment and create a fully customizable avatar that even includes facial expressions (the avatar feature did not exist at the time the intervention of this study was conducted). Users can invite other users to their digital home and interact. The Quest 2 also includes free immersive web-based multiplayer games that provide cooperative and competitive experiences through numerous recreational and social gaming experiences. Group VR recreation and socialization can likely provide an accessible opportunity for adolescents with disabilities to improve their physical fitness while building new friendships with peers that enhance their mental health.

This feasibility study aimed to explore the potential benefits and implementation processes of a digital VR program among adolescents with physical disabilities. This study had three purposes, which were to (1) examine the effects of a home-based VR multiplayer recreation and socialization on depression, socialization, and loneliness among 12 adolescents with physical disabilities; (2) quantify the acceptability of the program as measured by participant adherence, total play time, and exercise time; and (3) describe behavioral mechanisms that affected participant engagement.

## Methods

### Study Design

This study was a single group pre- to postdesign trial that lasted 1 year and included 2 waves. A wave was a cohort of 6 people who were assigned to complete the intervention together (n=6 per wave).

### Recruitment

Participants were recruited from the medical and billing record databases of the Children’s Hospital of Alabama. The study aimed to enroll a convenience sample of 12 people to satisfy minimum recommendations for a feasibility study, which aims to inform sample size considerations for a larger trial [21]. The eligibility criteria included (1) self-reported mobility disability (eg, the use of a mobility device or the presence of a mobility impairment), (2) between the ages of 13 and 19 years (World Health Organization definition of adolescence and the minimum age of 13 years as recommended by the manufacturer of the VR headset), (3) access to a Wi-Fi connection in the home, and (4) a caregiver to support the child if aged <18 years. The exclusion criteria were as follows: (1) physically active (defined as >150 min per week of moderate to vigorous intensity exercise), (2) cannot use the arms for exercise or operate the controller buttons using their fingers, and (3) complete blindness or deafness.

### Procedures

Interested participants were mailed the informed consent document and the study surveys. Participants were instructed to complete the consent forms and surveys if they wanted to join the study. Once the forms were received by the research staff, participants were mailed the intervention equipment. Participants were asked for their preferences for a time of day that they could likely attend the intervention. The intervention was scheduled at a time that was convenient for all participants for each of the 2 waves. Postintervention, participants were mailed another packet of surveys to complete and return to the research staff. Participants were then asked to participate in a one-on-one semistructured interview via phone call or Zoom (Zoom Technologies Inc) with the lead investigator (BL), which was audio recorded and transcribed for qualitative analysis. The interviews lasted approximately 30 minutes. A full description of the study procedures can be found elsewhere [22].

### Equipment

The equipment included a VR headset (now referred to as Meta Quest 2 vs Oculus Quest 2), which included handheld controllers. Upon receiving the headset, participants logged into the device with their personal Meta account. Research staff purchased games for participants through the Meta or Oculus website. Games were purchased as digital “gifts” to the participants, which participants redeemed digitally to their Meta account via an email activation link.

### Intervention

The intervention lasted 4 weeks and included 2×1-hour sessions per week of supervised peer-to-peer gaming. Participants used the Meta Quest headset to meet peers and 2 coaches (a gaming coach and a mindfulness coach) in a private party held digitally. Each session includes participation in 1 of the several games, including RecRoom (a massive multiplayer game with countless social and active gaming experiences), VRChat (a game focused on building social relationships), and Beat Saber (a rhythmic music-to-movement game). The coaches used behavioral change and mindfulness techniques to promote autonomy, competence, and relatedness through a respectful, cohesive, and positive atmosphere (strategies framed by the Self-Determination Theory [23-25] and learned from the mindfulness coaching workshops provided by the National Center for Health Physical Activity and Disability). Some of the mindfulness-based strategies included guided breathing–focused exercises, body scanning, meditation, and acceptance of social anxiety and shyness [26]. Some behavioral change strategies to promote active play included positive reinforcement for the successful completion of in-game activities and actions, reinforcement of a participation-focused atmosphere among peers as opposed to a focus on competition, as well as verbal reinforcement to promote high self-confidence. Participants were encouraged to add each other as a “friend” within the social platform of the headset and play together outside of class. Participants were instructed to be safe during web-based game, by not giving out any sensitive personal information, particularly to web-based players who were not part of the intervention classes.

### Measures

Aim 1 included self-report measures for (1) Children’s Depression Inventory 2 Short Form (CDI-S2), a measure of feelings of depression with strong psychometric properties among adolescents with and without disabilities [27,28] and (2) version 3 of the University of California, Los Angeles Loneliness Scale 20 items (UCLA-20) [29-31], a measure of social isolation and loneliness. The CDI-S2 includes 12 items that are scored from 0 to 2. Total raw scores are transformed into *T* scores based on the participant’s age and sex, using the scoring page attached to the instrument. A higher *T* score represents worsened feelings of depression. The UCLA-20 [29-31] is scored from 0 to 3 on each of the 20 items. Higher scores indicate worsened feelings of isolation and loneliness. The UCLA-20 version 3 is a measure of both loneliness and social isolation with strong psychometric properties among a variety of age groups and disability groups [31,32]. Descriptive statistics for all study outcomes (aims 1 and 2) included means, SD, effect sizes, box plots, and 95% CIs as appropriate. Aim 1 included *t* tests to compare pre- to postchanges in survey scores. Caregivers were instructed to assist the participants in completing the questionnaires.

Aim 2 was evaluated through the feasibility metrics such as participant attendance (percent of classes attended divided by the total, recorded by research staff), the type of games played during the group sessions, playtime with people outside of class friendships made and the strength of the friendships, and program satisfaction and enjoyment. Friendships were measured by a self-report survey that was created for this study, namely a Multiplayer Feedback Survey. The survey (Multimedia Appendix 1) was created to assess the frequency of play with peers outside of class, whether they made friends with peers and question the strength of these peer relationships, and to assess these same factors (play frequency, friendship creation, and friendship strength) among other players they met digitally outside of class. To measure program satisfaction, the research team used a satisfaction survey with 3 questions (Multimedia Appendix 2). Questions probed satisfaction with the social interactions, web-based group play, and how classes were conducted. The questions were scored on a 5-point Likert scale, with a score of 1 indicating “very dissatisfied” and a score of 5 indicating “very satisfied.” Similarly, enjoyment of the program was measured using a single-question score that pertained to the overall enjoyment of the program.

Aim 3 was measured through one-on-one semistructured interviews of participants’ perceptions of completing the program. The qualitative component of this study was a mini-ethnographic design [33,34], whereby an analyst (BL) was immersed in a culture with the participants and reported on the shared experience. Interviews were audio recorded and transcribed for analysis by a single analyst (BL). The analyst then coded the transcriptions and constructed categories or themes to represent the codes following a thematic analysis approach [34], which was underpinned by an interpretivist philosophical approach. The analyst’s (BL) ontological beliefs aligned with relativism (ie, the reality is multiple and subjective) and their epistemological beliefs with subjectivism (ie, knowledge is socially constructed) [34]. The thematic analysis process was guided by the 6 steps proposed by Braun and Clarke [35]. The analyst (BL) had several years of experience in qualitative research and has conducted over 400 interviews related to exercise and people with disabilities.

### Ethical Considerations

Prior to the enrollment of participants, this study was approved by the institutional review board of the university (FWA00005960). Recruitment occurred from August 2022 to January 2023. Written informed consent documentation was obtained from all participants prior to their engagement in the study. Participants received an electronic gift card that was loaded for US $60 for each packet of surveys returned (total of US $120 per completing the study).

## Results

### Participant Information

The mean age of participants was 16.6 (SD 1.8) years (male: n=9 and female: n=3). Eight participants were White (male: n=7 and female: n=1) and 4 were African American (male: n=2 and female: n=2). Three participants used a wheelchair as a primary means of mobility and 9 were ambulatory. A total of 9 participants had cerebral palsy, 2 had a spinal cord injury, and 1 had spina bifida. No adverse events were reported by participants.

### Process Metrics

Of the 12 enrolled participants, 4 (33%) participants dropped out of the program. One participant withdrew because of difficulty operating the controls and navigating the user interface. Two dropouts lost contact during the waiting period between receiving the equipment and waiting for the intervention to start; 1 completed the paperwork too late to attend the sessions (after both waves were completed). Mean attendance for the remaining 8 participants was 77% (49 sessions of the 64 total possible sessions; mean 6, SD 2 sessions).

### Resource and Management Metrics

The duration required to mail the surveys to participants and receive the completed surveys from each participant wave took approximately 3.5 weeks. Shipping the headsets to each participant wave and preparing them for the group sessions (redeeming the purchased games and adding the instructors as friends within the Oculus social platform) took approximately 2 weeks.

Regarding study supplies, 1 participant reported that they did not receive the headset, despite package tracking stating that the headset was delivered. This participant was shipped another headset. Of note, this participant was lost to contact after the second headset was shipped. The study budget included the capacity to purchase a maximum of 4 games per person, and this was sufficient to complete the intervention.

**Figure 1:**
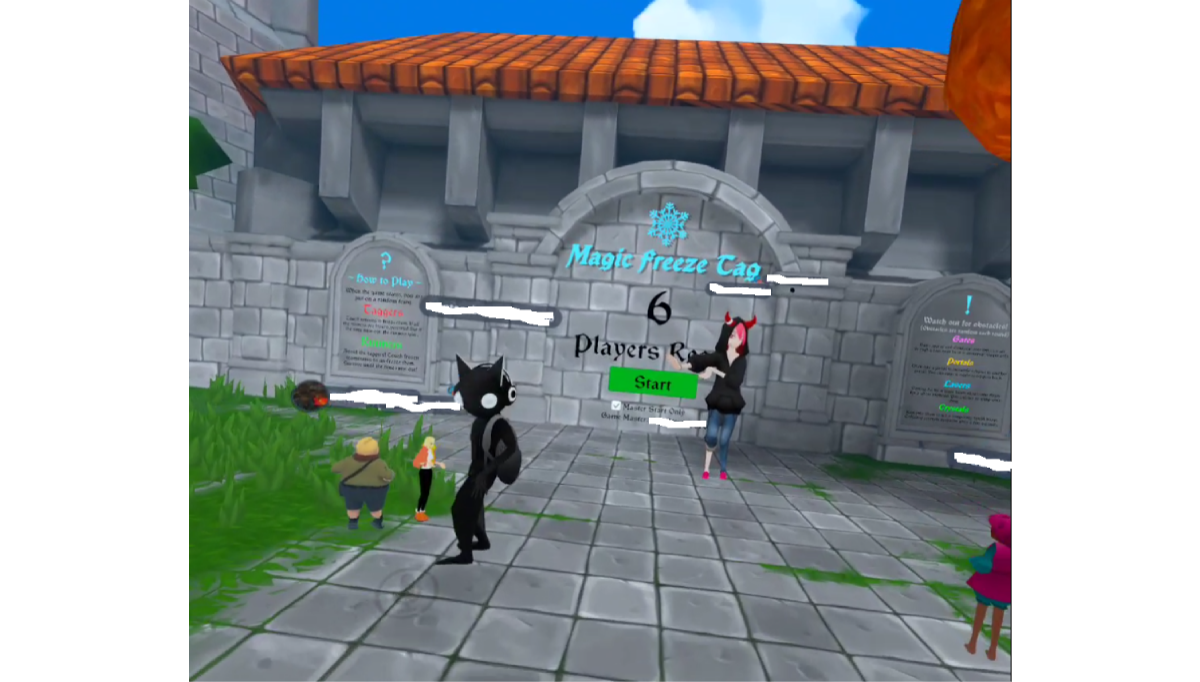
View of a the waiting room of an intervention game (freeze tag) in VRChat for participants with disabilities.

**Figure 2:**
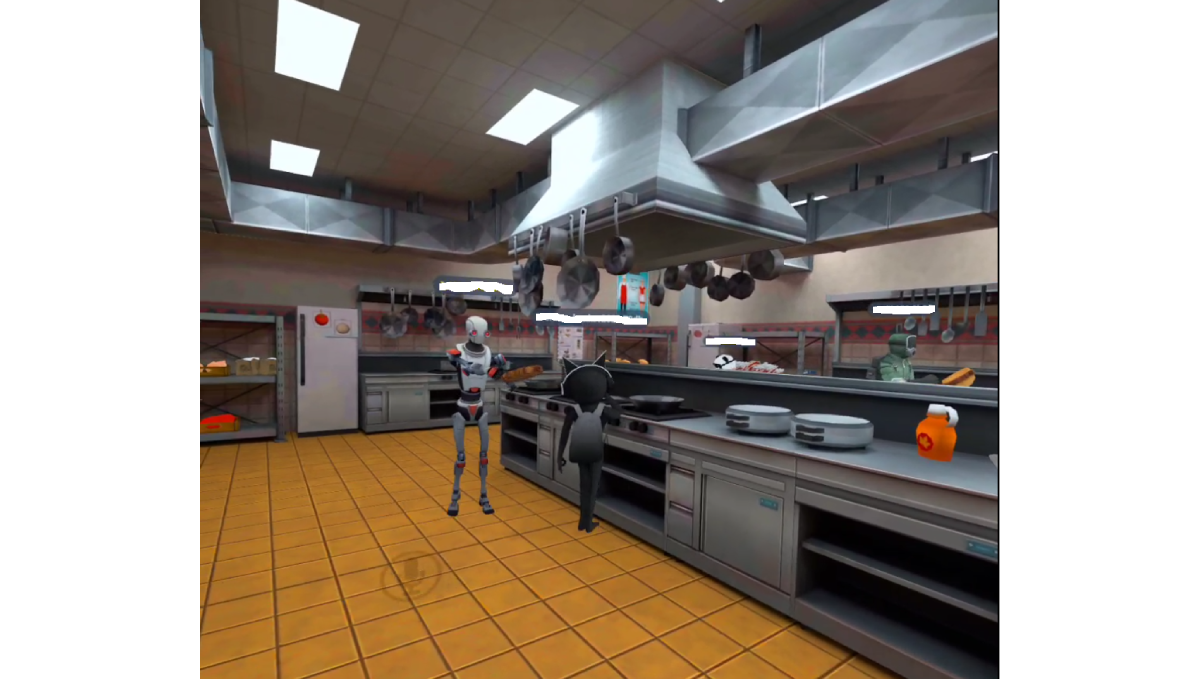
View of a cooperative kitchen cooking scenario for a group of participants with disabilities in VRChat.

**Figure 3:**
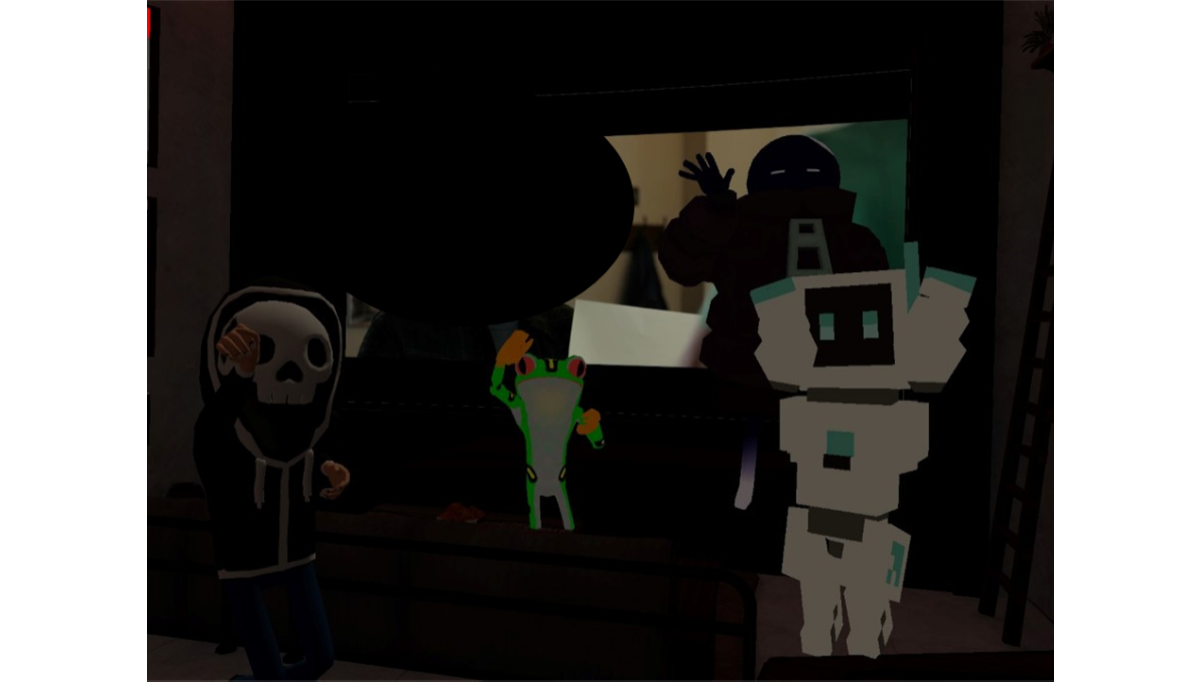
View of a group of participants watching a movie in virtual reality in the game VRChat.

During wave 1, the research team learned that the best group-based game was VRChat, followed by Dash Dash World, Rec Room, and Beat Saber. VRChat could be downloaded at no cost and included numerous games and social experiences. VRChat was by far the most played game. VRChat was the easiest game to operate for group play because the Oculus social app included a “GO” button that could launch the entire group into the same location. Most games and experiences within VRChat had a maximum player capacity that far exceeded the group size of 8 (2 instructors and 6 participants). Most other multiplayer games had a maximum capacity of either 2 or 4 players or required complex controller skills that exceeded the ability of some participants within each wave. During wave 2, the Oculus social platform was updated with the ability to have digital avatars that could be edited to look like the player. The avatars included a torso, head, and arms, along with facial expressions, mouth movements, and eyes that would seemingly react to the player’s speech. Player avatars met in the instructor’s digital home, and this was the primary location for starting the class and socializing in wave 2. The home environment could be changed to a variety of different digital settings. In wave 1, most participants seemingly enjoyed experiences that emphasized gameplay (eg, racing [VRChat and Dash Dash World] and freeze tag [VRChat] as shown in Figure 1 and cooperative cooking [VRChat] as shown in Figure 2). Wave 2 participants preferred listening to music and hanging out in VRChat (Figure 3). The Oculus social platform required an Oculus app to be downloaded on a mobile phone. This mobile app was used by participants to communicate with each other and schedule out-of-class play sessions.

### Scientific Outcomes

#### Overview

All 8 participants who completed the intervention also completed the postintervention surveys.

#### UCLA-20 and CDI-S2

**Figure 4:**
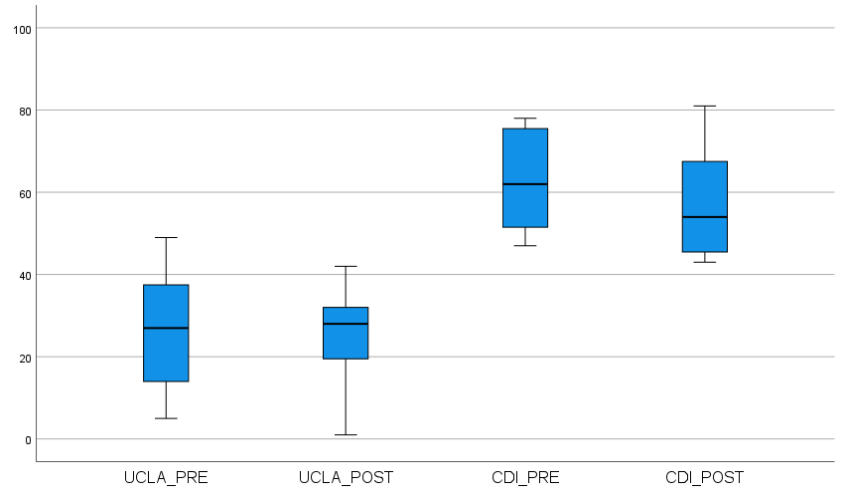
Box plots of survey results at pre (week 0) and post intervention (week 5). The y-axis represents the score of each survey. UCLA values are raw scores. CDI values are *z* scores to for better visual representation. CDI: Children’s Depression Inventory; UCLA: University of California, Los Angeles.

Paired *t* tests (2-tailed) demonstrated no significant difference between pre- and postintervention scores for the UCLA-20 (mean preintervention score 26.4, SD 15.4; mean postintervention score 25.3, SD 12.4; *P*=.82; effect size=0.08, 95% CI –0.614 to 0.774). Five participants reported an improvement in the UCLA-20 score. Three participants reported a worsened UCLA-20 score, 1 of whom reported an outlying increased score (poorer state of isolation and loneliness) of 25 points. When this participant was asked for the reason for a score increase, the person noted that the increased score was due to “drama” at school. Paired *t* tests for the CDI-S2 scores demonstrated a trend of improved scores (mean preintervention score 7.25, SD 4.2; mean postintervention score 5.38, SD 4.1; *P*=.06; effect size=0.45, 95% CI –0.15 to 3.9), but this was not statistically significant. Five participants reported improved scores, 2 reported worsened scores, and 1 reported no change. Mean *z* scores for the CDI-S2 were 62.9 (SD 12.6) at preintervention and 57.3 (SD 14.1) at postintervention. Box plots for the UCLA-20 and CDI-S2 are displayed in Figure 4.

#### Multiplayer Feedback Survey

Regarding the multiplayer feedback survey, 50% (4/8) of participants reported that they played with a peer outside of the class sessions. One reported that they played with a peer 3 to 4 times per week outside of class, and the other 3 reported that they played with a peer 1-2 times per week. Most participants (7/8, 88%) stated that they became friends with a peer in class. The strength of the peer friendship was reported as “friends” (2 participants), “growing friendship” (2 participants), “someone to relate to” (1 participant), and “not strong” (2 participants). Additionally, 6 (75%) participants reported that they played with other people outside of the group class. The frequency of play was reported as “>4-5 times per week” by 1 participant, “3-4 times per week” by 1 participant, and “1-2 times per week” by 4 participants. Furthermore, 4 (50%) participants reported that they established friendships with other people they met digitally. The strength of friendship was reported as “strong friendship/best friends” by 1 participant, “friends” by 1 participant, “growing a friendship” by 1 participant, and “not strong” by 2 participants.

#### Satisfaction and Enjoyment

Mean satisfaction with the social interactions during VR gameplay resulted in a score of 4.75 (SD 0.46), satisfaction with web-based group play was 4.5 (SD 1), satisfaction with how the classes were conducted by instructors was 5 (SD 0), and enjoyment was 4.86 (SD 0.46).

#### Qualitative Themes

##### Overview

The analysis resulted in four themes: (1) user experience dictated game performance and preference, (2) enjoyable gaming and meaningful social interactions determined attendance, (3) web-based communications attenuated real-world social isolation, and (4) barriers to participation. The themes are summarized below and displayed in Textbox 1.

**Qualitative themes of participant perspectives after completion of the study.**

**User experience dictated game performance and preference**

- Participants frequently played video games during their leisure time.
- Experienced gamers preferred active and complex recreational games during class, whereas less experienced gamers preferred sessions that emphasized socialization versus gameplay.
- Issues with headset and controller operation on some games impeded performance and attendance.
- Personal video game interests influenced engagement level during the class sessions.

**Attendance determined by enjoyable gaming and meaningful social interactions**

- Participants looked forward to the sessions because the sessions were perceived as fun.
- Participant motivated caregiver to stay on top of the program.
- Games were perceived as enjoyable.
- Participants were motivated to attend due to social experiences in virtual reality.
- Headset increased communication with family members (group play and shared play).
- The deeper the perceived bond between peers the better the attendance at the sessions.
- Participants with high attendance found another class member who they could relate with.

**Web-based communications attenuated real-world social isolation**

- Some participants in each intervention wave were highly active in contacting peers through SMS text messaging.
- The participant does not have many close friends in school.
- Participants bullied at school or related people at school with more opportunities to create drama.
- Participant’s closest friends are family members.
- Participants enjoyed spending time with peers with disabilities.
- Participants reported isolation from outdoor activities due to being immunocompromised.
- Participants enjoyed the interactions with other people because they are used to social isolation—feelings of shyness due to fear of interactions.
- Participant perceives feeling “out of place” with other people their age at school due to their disability.
- Caregivers acknowledge a need for their child to socialize with peers.

**Barriers to participation**

- The participant reported being shy at the beginning of the program but became more comfortable with communicating near the end of the program.
- College or schoolwork interfered with class attendance for participants with low adherence.
- Class time was perceived as inconvenient which contributed to nonattendance.
- Families who were not familiar with video games or video game technology recommended additional instruction at the beginning of the program.
- Some chosen games were not fully usable for people with operation of one hand.

##### User Experience Dictated Game Performance and Preference

The program included 2 participants who had 0 video game experience, along with 6 participants with several years of video game experience. Experienced video gamers quickly adapted to the handheld controllers and, thus, preferred class sessions with complex cooperative and competitive gameplay, whereas inexperienced gamers preferred games that focused more on social experiences and minimal controller operation. Personal video game interests (eg, racing and first-person shooters) also influenced engagement in the class sessions.

##### Attendance Determined by Enjoyable Gaming and Meaningful Social Interactions

The program was highly praised for being enjoyable due to the social experiences that were created while immersed in a video game. Participants looked forward to attending the program to play with peers, family members, and even other players they met through web-based play. Participants who had high attendance to the class sessions had a strong friendship bond with other peers in the class.

##### Web-Based Communications Were Sought to Attenuate Real-World Isolation

Participants desired to join the program to mitigate feelings of social isolation that were experienced during everyday life. Participants perceived that their disability made them feel out of place at school. They reported a few close friends who were not family members. Two participants reported that they participate in digital school at home. Some participants did not participate in outdoor activities due to concerns about how COVID-19 would impact people who are immunocompromised. Moreover, parents were highly supportive of enrolling their children in the program. Parents acknowledged that their children greatly needed social opportunities. Thus, recruitment for this study was not difficult. The instructors found that prompting participants to communicate with peers outside of the class required very little encouragement. Enjoyment was a frequent term that participants used to describe their time with peers in the program. Outside of the class, participants established friendships through text messaging and scheduled meetups in a VR game.

##### Barriers to Participation

Although some participants were quick to attempt to contact their peers outside of class, some participants reported that feelings of shyness prevented them from building meaningful relationships with peers. Feelings of shyness were strongest at the start of the program. Participants with special needs (eg, intellectual disability, issues with dexterity, or visual issues) or families who were not comfortable with the technology required additional support to learn how to operate the controller and user interface. Some games and activities were not fully usable for participants who could not independently operate the controls with sufficient speed or coordination. Additionally, finding a class time that was convenient for all members was difficult. Some participants had schoolwork or family activities that conflicted with their attendance in the classes.

## Discussion

### Principal Findings

This study tested a group-based VR program to improve mental health among adolescents with mobility disabilities using an off-the-shelf VR gaming headset. The study findings demonstrated a potential effect on mental health and acceptable feasibility through several metrics: attendance of 77% (49/64 sessions), high levels of perceived enjoyment and satisfaction, and participants connected with peers outside of class or with other web-based players and built relationships of varying strengths.

Although VR has been used in a variety of clinical settings for symptoms management (eg, pain and anxiety) [36,37], to the best of our knowledge, this study was the first to test the Meta Quest 2 as a home-based method for web-based group socialization among adolescents with physical disabilities. The implementation of the intervention was successful. However, the preintervention preparation phase was notably more difficult than anticipated (duration of ~3.5 weeks). Synchronizing 6 adolescents to complete and return the questionnaires and start the intervention at the same time was challenging, due to families having limited availability amid a complex daily routine (eg, school activities, medications, meals, homework, self-care, and therapy). Two participants dropped out because the study staff lost contact with them at this phase. They could not be reached after 3 consecutive phone calls. One solution to address this could be ongoing enrollment instead of enrollment waves—a more flexible intervention that can permit participants to join the program as soon as they complete their preparation work (survey completion and headset setup). To enhance the effects of the program on mental health, a web-based VR program could include a licensed pediatric psychologist to incorporate more complex strategies for improving mental health during group play and socialization.

Qualitative study findings identified behavioral mechanisms that underpinned participants’ engagement in the program. Adolescents with disabilities were highly motivated to join the trial, but motivations for joining the trial differed. Participants were highly motivated to try the VR headset, which was due to their experience with video games, their curiosity about VR technology, and their desire to interact with peers with disabilities. Their level of engagement (ie, attendance) was determined by the level of fun obtained from the games, their level of interactions and bonds with peers with disabilities, negative interactions with peers at school, and school responsibilities. Caregivers were motivated by the perception that their child needed opportunities for socialization, as well as by the child. Therefore, future trials that implement this type of intervention need not worry about enrollment but should focus on strategies that maximize attendance.

The complexities of participant preferences could be addressed in more complex intervention designs. Participant preference and expertise with video games were critical factors of program engagement. Intervention wave 1 was notably different from wave 2. Wave 1 included more people who preferred group gameplay and recreation, whereas wave 2 had people who mostly enjoyed socialization in different digital environments. One solution could be to use an adaptive intervention design [38], where engagement factors dictate the type and dose of intervention that a participant is prescribed, as well as how the intervention is modified while ongoing. For example, in an adaptive intervention design, there could be 2 intervention options: 1 focused on gameplay and 1 focused on socialization. Participants could be allocated to an intervention option based on their preference and receive mindfulness coaching based on their individualized needs. An adaptive design allows flexibility to meet individualized needs while maintaining research rigor through a unique control group design. Nevertheless, such designs will require large sample sizes to ensure adequate comparisons between multiple groups.

### Limitations

The sample size of this feasibility study prevented the generalization of the study findings but will inform sample size considerations for a confirmatory randomized controlled trial. The lack of a control group hinders the confirmability of study findings. Another limitation was that this study did not record the minutes of play for each game that was played during the session. Manual recording of gameplay minutes was difficult since the research staff wore a headset during the intervention. At the time the study was completed, the study team was not aware of software that allowed built-in monitoring of gameplay minutes. Future studies could use a recent advancement in software, “Oculus Move,” which is now preinstalled within all Quest VR headsets. This software allows minutes of gameplay to be recorded and viewed for the previous month. Furthermore, the feasibility metrics of this study were superficial. In retrospect, measures could have been included to assess gaming aspects of the intervention, for example, gaming engagement [39] and immersion [40].

### Conclusions

The study findings demonstrated that immersive web-based VR could be used to promote relationship building among adolescents with physical disabilities. Group-based VR may also be a channel for promoting mental health. Study findings identified factors that can be addressed to enhance implementation processes, effectiveness, and participant engagement of the program in a larger clinical trial.

## Appendix

Multimedia Appendix 1 Multiplayer feedback survey.

Multimedia Appendix 2 Satisfaction with program delivery.

## Abbreviations

CDI-S2: Children’s Depression Inventory 2 Short Form
HMD: head-mounted display
VR: virtual reality
UCLA-20: University of California, Los Angeles Loneliness Scale 20 items

## Data Availability

The data sets generated and analyzed during this study are available from the corresponding author on reasonable request.
