# Supplementary material for: Improving Social Isolation and Loneliness Among Adolescents With Physical Disabilities Through Group-Based Virtual Reality Gaming: Feasibility Pre-Post Trial Study
Source: JMIR Form Res. 2023 Dec 6;7:e47630. doi: 10.2196/47630 (PMC10733831; doi:10.2196/47630)
Supplement: Multimedia Appendix 1 [file formative_v7i1e47630_app1.docx]

Multiplayer feedback survey

1. Did you play with peers outside of class? If yes, circle yes, and how many times per week?

| 1  Yes | 2  No |
| --- | --- |

If “Yes” above, How many times per week? If “No”, skip below to question 2.

| 1  None | 2  1-2 times per week | 3  2-3 times per week | 4  3-4 times per week | 5  > 4-5 times per week |
| --- | --- | --- | --- | --- |

2. Have you become friends with any of your peers in the group class? If yes, circle “Yes”, and describe how strong these friendships are. If “No”, skip to question 3.

| 1  Yes | 2  No |
| --- | --- |

If “Yes” above, describe how strong these friendships are. If “No”, skip below to question 3.

| 1  Strong Friendships/ Best Friends | 2  Friends | 3  Someone to relate to | 4  Growing a friendship | 5  Maybe in the future, but not right now |
| --- | --- | --- | --- | --- |

3. Did you play with other people outside of the class online? If “Yes”, circle “Yes”, and how many times per week?


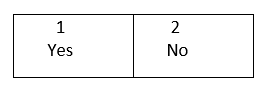


If “Yes” above, How many times per week? If “No”, skip below to question 4.


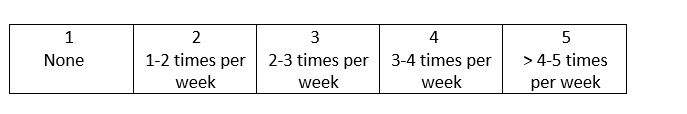


4. Outside of the group class, did you establish other friendships with other online people?

| 1  Yes | 2  No |
| --- | --- |

If “Yes” above, describe how strong these friendships are. If “No”, you have completed the multiplayer feedback survey.

| 1  Strong friendships/ Best Friends | 2  Friends | 3  Someone to relate to | 4  Growing a friendship | 5  Maybe in the future, but not right now |
| --- | --- | --- | --- | --- |
