# Supplementary material for: Improving Social Isolation and Loneliness Among Adolescents With Physical Disabilities Through Group-Based Virtual Reality Gaming: Feasibility Pre-Post Trial Study
Source: JMIR Form Res. 2023 Dec 6;7:e47630. doi: 10.2196/47630 (PMC10733831; doi:10.2196/47630)
Supplement: Multimedia Appendix 2 [file formative_v7i1e47630_app2.docx]

Satisfaction with program delivery

For each question, please circle your best answer choice.

**How satisfied were you with the social interaction during virtual reality game play?**

1. Very dissatisfied

2. Moderately dissatisfied

3. Neither satisfied nor dissatisfied

4. Moderately satisfied

5. Very satisfied

**How satisfied were you with the online group play?**

1.Very dissatisfied

2. Moderately dissatisfied

3. Neither satisfied nor dissatisfied

4. Moderately satisfied

5. Very satisfied

**How satisfied are you overall with how the classes were conducted by the instructors?**

1. Very dissatisfied

2. Moderately dissatisfied

3. Neither satisfied nor dissatisfied

4. Moderately satisfied

5. Very satisfied

**Overall, how enjoyable were the group classes?**

1. Very dissatisfied

2. Moderately dissatisfied

3. Neither satisfied nor dissatisfied

4. Moderately satisfied

5. Very satisfied
